# THE IMPACT OF FAM-FFC UPON ADVERSE OUTCOMES IN HOSPITALIZED PERSONS WITH DEMENTIA

**DOI:** 10.1093/geroni/igad104.0303

**Published:** 2023-12-21

**Authors:** Barbara Resnick

**Affiliations:** University of Maryland, Baltimore, Maryland, United States

## Abstract

The Fam-FCC education and coaching of care partners emphasizes optimizing physical and cognitive function and detection of changes in condition to prevent falls, avoidable transfers to the emergency department and hospital readmissions. Thus, the aim of this study was to examine the impact of Fam-FFC upon the desired outcomes of less transfers to the emergency department, hospital readmissions, and falls. The majority of patients identified as female, with an average age of 81.5 (± 8.4); approximately 64% were White patients and 36% were Black or African American patients. The sample indicated, on average, moderate impairment in cognition (MMoCA=11.7±7.0) and baseline (pre-admission) some impairment in physical function (MBI=78.1±22.6), mild pain (MPAINAD=0.79±1.5) and depression (MCESD=9.1±6.2). There were no group differences in transfers to the emergency department; however intervention participants were approximately 10% less likely to be hospitalized (p<.04) over the study intervention. The total number of falls or transfers to the emergency department did not differ between groups. The findings of this study suggest that Fam-FFC may be implemented without increasing the risk of falls, while possibly decreasing hospitalizations.

